# Prevalence and Frequency of Self-measured Blood Pressure Monitoring in US Adults Aged 50-80 Years

**DOI:** 10.1001/jamanetworkopen.2022.31772

**Published:** 2022-09-14

**Authors:** Mellanie V. Springer, Preeti Malani, Erica Solway, Matthias Kirch, Dianne C. Singer, Jeffrey T. Kullgren, Deborah A. Levine

**Affiliations:** 1 Department of Neurology, University of Michigan, Ann Arbor; 2 Institute for Healthcare Policy and Innovation, University of Michigan, Ann Arbor; 3 Division of Infectious Diseases, Department of Internal Medicine, University of Michigan, Ann Arbor; 4 Child Health Evaluation and Research Center, University of Michigan, Ann Arbor; 5 Center for Clinical Management Research, Veteran Affairs Ann Arbor Healthcare System, Ann Arbor, Michigan; 6 Department of Internal Medicine, University of Michigan, Ann Arbor; 7 Department of Health Management and Policy, University of Michigan, Ann Arbor; 8 Division of General Medicine, Department of Internal Medicine, University of Michigan, Ann Arbor

## Abstract

This survey study investigates the prevalence, frequency, and factors associated with self-measured blood pressure monitoring among adults ages 50 to 80 years.

## Introduction

Guidelines recommend that patients use self-measured blood pressure monitoring (SBPM) outside the clinic to diagnose and manage hypertension.^1^ The current prevalence and frequency of SBPM in the US are unclear.^2^ This study estimated prevalence, frequency, and factors associated with SBPM in US adults aged 50 to 80 years with hypertension or a blood pressure–related health condition (BPHC).

## Methods

The University of Michigan institutional review board deemed this survey study exempt from full review because respondents were deidentified and waived informed consent because participants were selected from an existing online volunteer panel of respondents. The survey response rate is reported following the AAPOR reporting guideline.

The University of Michigan National Healthy Poll on Aging^3^ was conducted in January 2021. Respondents aged 50 to 80 years were randomly selected from KnowledgePanel (Ipsos Public Affairs), a probability-based sample weighted to March 2020 US Census Bureau Current Population Survey measures of sex, age, race and ethnicity, education, region, household income, home ownership, and metropolitan area. Weights were adjusted to account for differential nonresponse.^4^

Respondents reported current antihypertensive medication use and histories of hypertension and BPHC, defined as stroke, coronary heart disease, myocardial infarction, congestive heart failure, diabetes, and chronic kidney disease. Prevalence of regular SBPM was asked of all respondents. Respondents who owned an SBPM device were asked about prevalence of regular SBPM via frequency of use, reasons for SBPM, and clinician engagement with SBPM (Table).

**Table.** Weighted Percentage of Responses, Participant Characteristics, and Logistic Regression Outcomes

Metric	With hypertension or BPHC (n = 1247)	With hypertension (n = 1050)
**Question, % (95% CI)^a^**		
Have you ever been told by a doctor, nurse, or another health professional to periodically check your blood pressure outside of the health care system?)		
Respondents, No.	1245	1048
Yes	61.6 (58.6-64.5)	68.2 (65.0-71.2)
No	38.4 (35.5-41.4)	31.8 (28.8-35.0)
Do you regularly monitor your own blood pressure?		
Respondents, No.	1242	1046
Yes	47.9 (44.9-50.9)	51.2 (47.9-54.4)^b^
Why do you monitor your blood pressure? Select all that apply^c^		
Respondents, No.	608	551
To be as healthy as possible	59.8 (55.5-64.0)	61.0 (56.5-65.4)
Because my doctor suggested it	50.8 (46.4-55.1)	52.5 (48.0-57.0)
To prevent a decline in my cognitive function	13.5 (10.8-16.7)	13.5 (10.8-16.9)
To reduce my risk of stroke	37.4 (33.2-41.7)	37.2 (32.9-41.7)
To reduce my risk of kidney disease	17.1 (14.0-20.8)	16.8 (13.6-20.5)
To reduce my risk of heart disease	31.9 (28.0-36.1)	31.9 (27.8-36.3)
Do you have a home blood pressure monitor device with an arm cuff?		
Respondents, No.	1247	1050
Yes, and I use it	55.0 (52.0-58.0)	58.7 (55.4-61.9)
Yes, but I don’t use it	19.1 (16.8-21.5)	18.0 (15.6-20.6)
No	26.0 (23.4-28.8)	23.3 (20.6-26.3)
Do you share your home blood pressure readings with a doctor, nurse, or another health professional?^d^		
Respondents, No.	702	632
Yes	50.2 (46.2-54.3)	51.5 (47.3-55.7)
No	49.8 (45.8-53.8)	48.5 (44.3-52.8)
Does your doctor, nurse, or another health professional provide feedback on your home blood pressure readings?^e^		
Respondents, No.	351	326
Yes	88.4 (84.3-91.6)	90.1 (86.1-93.0)
No	11.6 (8.5-15.7)	9.9 (7.0-13.9)
Why do you not have a home blood pressure monitor device?^f^		
Respondents, No.	302	224
Too expensive	16.0 (11.8-21.3)	18.0 (13.0-24.4)
Not able to find one	0	0
Don’t think I need one/never thought about getting one	54.0 (47.9-60.1)	47.0 (40.0-54.1)
Not sure how to use it/too complicated	4.6 (2.8-7.5)	4.7 (2.6-8.4)
Don’t think they’re accurate	7.6 (5.0-11.4)	10.1 (6.7-15.0)
Other	22.4 (17.7-28.0)	24.9 (19.2-31.5)
**Demographic characteristic, No. (% [95% CI])** ^g^		
Age, mean (SD), y	64 (0.26)	NC
Sex		
Men	632 (51.0 [48.0-54.0])	NC
Women	615 (49.0 [46.0-52.0])	NC
Race and ethnicity		
Black	133 (12.3 [10.3-14.5])	NC
Hispanic	105 (11.0) [9.1-13.3])	NC
White	938 (70.9 [67.9-73.7])	NC
Other	71 (5.8 [4.4-7.7])	NC
Education		
High school	423 (41.4 [38.4-44.5])	NC
Some college	432 (30.3 [27.7-33.0])	NC
Bachelor’s degree	392 (28.3 [25.8-31.0])	NC
Clinical characteristic		
Hypertension	1050 (84.4 [82.1-86.4])	NC
Stroke	89 (6.9 [5.5-8.7])	NC
Coronary artery disease	145 (11.6 [9.8-13.6])	NC
Myocardial infarction	89 (7.3 [5.8-9.0])	NC
Congestive heart failure	71 (5.9 [4.6-7.5])	NC
Diabetes	475 (39.2 [36.3-42.2])	NC
Chronic kidney disease	93 (7.5 [6.0-9.3])	NC
**Odds of regular SBPM, OR (95% CI)** ^h^		
Age 65-80 y (reference: age 50-64 y)	0.99 (0.70-1.39)	NC
Women	0.99 (0.70-1.38)	NC
Race and ethnicity (reference: White)		
Black	1.81 (0.99-3.29)	NC
Hispanic	1.40 (0.84-2.36)	NC
Other	1.56 (0.71-3.43)	NC
Self-rated mental health (reference: fair or poor)		NC
Excellent or good	2.02 (1.03-3.98)	NC
Good	1.17 (0.58-2.35)	NC
Clinician recommendation to perform SBPM	3.51 (2.43-5.07)	NC
Own a home BP monitor with an arm cuff (reference: no)		
Yes, and I use it	10.75 (7.28-15.8)	NC
Yes, but I do not use it	0.15 (0.06-0.34)	NC
Currently using medication to control high BP	1.44 (0.96-2.15)	NC

Abbreviations: BP, blood pressure; BPHC, blood pressure–related health condition; NC, not calculated; SBPM, self-measured blood pressure monitoring.

^a^ Questions are those asked verbatim on the survey.

^b^ All respondents with a diagnosis of hypertension. Among respondents with a diagnosis of hypertension who did not own a home blood pressure cuff, 21.4% (95% CI, 16.3%-27.5%) reported regularly monitoring their own blood pressure.

^c^ Asked only if the answer to the previous question in the table was yes. Multiple responses allowed. Response options were generated by author consensus.

^d^ Asked only of individuals who used a home blood pressure monitoring device to check their blood pressure. The yes category collapses the following response options “Yes, I take them to my doctors visits”; “Yes, my blood pressure device automatically reports them to my doctor”: and “Yes, I send them to my doctor.”

^e^ Asked only if the answer to the previous question in the table was yes.

^f^ Asked only if the answer to the question, “Do you have a home blood pressure monitor device with an arm cuff?” was no. Multiple responses allowed.

^g^ Percentages are rounded and therefore may not add to 100. All percentages are weighted to reflect population estimates.

^h^ ORs were considered significant when 95% CIs did not cross 1 (ie, at *P* < .05).

The primary outcome was regular SBPM (yes or no). Outcome and weighted responses to survey questions were examined among respondents with hypertension or a BPHC and those with hypertension only because they warrant blood pressure control. Logistic regression was done using Stata statistical software version 15.1 (StataCorp) to examine regular SBPM before and after adjusting for participant factors (eMethods in the Supplement).

## Results

Of 2583 adults requested to participate, 2023 individuals (78.0%) completed the survey; among them, 1247 individuals (59.6% [95% CI, 57.2%-61.9%]) had hypertension or a BPHC (mean [SD] age, 64.19 [0.26] years; 632 [weighted percentage, 51.0%] men) (Table) and 1050 individuals (84.4%[95% CI, 82.1%-86.4%]) had hypertension only. Among those with hypertension or a BPHC, 61.6% (95% CI, 58.6%-64.5%) reported that their clinicians advised them to periodically check their BP (Table) and 47.9% (95% CI, 44.9%-50.9%) of participants reported that they regularly monitor their BP. Of respondents with hypertension, 51.2% (95% CI, 47.9%-54.4%) regularly monitored their BP. The Table presents reasons for performing SBPM. Among adults with hypertension or BPHCs, 55.0% (95% CI, 52.0%-58.0%) owned and used a home BP monitor at different frequencies (Figure). Among them, approximately half shared home BP readings with their clinicians, and most individuals received feedback from clinicians (Table). Of respondents who did not own a monitor (26.0% [95% CI, 23.4%-28.8%] of respondents), 54.0% (95% CI, 47.9%-60.1%) reported that they did not think they needed or never thought about getting one (Table). Self-rated mental health, clinician recommendation, and home SBPM device ownership were associated with regular SBPM, but no other factors were (Table).

**Figure.**
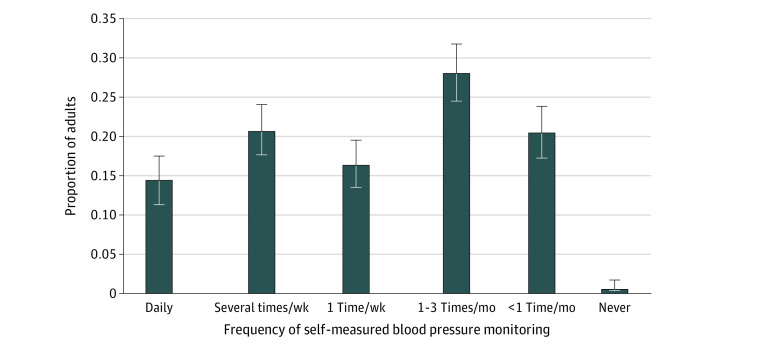
Frequency of Self-measured Blood Pressure Monitoring Surveyed among adults with hypertension and blood pressure–related health conditions who own a home blood pressure monitor.

## Discussion

In this survey study among a nationally representative sample of adults aged 50 to 80 years, less than half of those with hypertension or BPHCs regularly monitored BP. SBPM prevalence of those with hypertension in 2021 (51.2%) was higher than among US adults with hypertension aged 18 years or older in 2005 to 2008 (43%), which is perhaps associated with our sample’s older age.^5^ Indeed, this study uniquely reports SBPM prevalence in adults ages 50 to 80 years with hypertension or BPHCs, who have a higher risk of adverse outcomes from uncontrolled BP than younger adults.

More adults in this study compared with the National Health and Nutrition Examination survey^6^ reported a clinician recommendation for SBPM. The decrease of in-person office visits with increased telehealth may be associated with increasing clinician recommendations to perform SBPM. Study limitations include use of self-reported data, that regular SBPM does not refer to a specific BP monitoring frequency, and limited representation of racially and ethnically underrepresented groups.

Home BP monitoring is associated with moderate decreases in BP and is cost-effective.^2^ Our results suggest that protocols should be developed to educate patients about the importance of SBPM and sharing readings with clinicians and the frequency that SBPM should be performed.

## Notes

Supplement.
eMethods.
