# Supplementary material for: Prevalence and Frequency of Self-measured Blood Pressure Monitoring in US Adults Aged 50-80 Years
Source: JAMA Netw Open. 2022 Sep 14;5(9):e2231772. doi: 10.1001/jamanetworkopen.2022.31772 (PMC9475387; doi:10.1001/jamanetworkopen.2022.31772)
Supplement: Supplement. — eMethods. [file jamanetwopen-e2231772-s001.pdf]

Supplemental Online Content

Springer MV, Malani P, Solway E, et al. Prevalence and frequency of self-measured blood pressure monitoring in US adults aged 50-80 years. *JAMA Netw Open*. 2022;5(9):e2231772. doi:10.1001/jamanetworkopen.2022.31772

eMethods.

This supplemental material has been provided by the authors to give readers additional information about their work.

© 2022 Springer MV et al. *JAMA Network Open.*

eMethods

We used chi-square analyses to estimate the association between regular SBPM (yes/no) and age (50-64 and 65-80 years old), sex (male or female), race and ethnicity (White, Black, Hispanic, and Other), self-rated physical and mental health (excellent or very good, good, fair or poor), provider recommendation to perform SBPM (yes/no), home SBPM monitor device ownership (yes and I use it/yes but I don't use it/no), and antihypertensive medication use (yes/no). Race and ethnicity were self-reported. Races on the US Census bureau current population survey included Asian, American Indian or Alaska Native, Black or African American, Native Hawaiian or other Pacific Islander, and White. Participants were permitted to report more than 1 race. Ethnicity was determined by asking, "Are you Hispanic, Latino, or Spanish origin?" Response options included yes or no. Respondents were not told that there was an other race category. If the respondent reported a race that was not on the list of races, then the survey administrator coded the reported race as other. Race and ethnicity were asked as part of demographic data. For survey weights, the race and ethnicity categories were reclassified as Hispanic, non-Hispanic Black, non-Hispanic White, non-Hispanic other, and non-Hispanic with 2 or more racial identities. Self-rated physical and mental health were assessed by the survey question "In general, how would you rate your physical [mental] health?" Response options included excellent, very good, good, fair, or poor. The dichotomous outcome of regular SBPM was used as we did not have information about respondents' hypertension control, which guides the recommended frequency of performing SBPM. The logistic regression model included demographics and participant factors (listed above) significantly associated with regular SBPM at a p-value<0.05. Missing observations were dropped from the analysis (<0.5% of refusals for each item used in this analysis). Analysis was performed using survey weights.

© 2022 Springer MV et al. *JAMA Network Open.*
